# Supplementary material for: Global phosphoproteomic analysis reveals ARMC10 as an AMPK substrate that regulates mitochondrial dynamics
Source: Nat Commun. 2019 Jan 10;10:104. doi: 10.1038/s41467-018-08004-0 (PMC6328551; doi:10.1038/s41467-018-08004-0)
Supplement: Supplementary file 6 — Description of Additional Supplementary Files [file 41467_2018_8004_MOESM6_ESM.pdf]

Description of Additional Supplementary Files

File Name: Supplementary Data 1

Description: Dataset of the phosphoproteomics data. All identifications from four biological repeats listed in the sheet, including fold changes and p values (Student's t-test). The peptides with two or more phosphorylation sites were treated as two or more different phosphopeptides.

File Name: Supplementary Data 2

Description: Differential phosphosites lists. The data were filtered from Table S1 by selecting those peptides whose fold-change in phosphorylation between wild-type (WT) cells and AMPK□1/2-DKO cells was greater than 1.5 and p value lower than 0.01 (t-test).

Sheet 1. 109 phosphosites with higher phosphorylation levels in WT cells than in AMPKα1/α2-DKO cells.

Sheet 2. 51 phosphosites with higher phosphorylation levels in AMPKα1/α2-DKO cells than in WT cells.

File Name: Supplementary Data 3

Description: Functional analysis for Fig. 2c. The functional annotation details for Fig. 2c. This table includes all the proteins listed in the three groups.

File Name: Supplementary Data 4

Description: Novel AMPK substrate phosphosites. The 24 novel AMPK substrate phosphosites identified by this analysis.
