# Supplementary material for: Global phosphoproteomic analysis reveals ARMC10 as an AMPK substrate that regulates mitochondrial dynamics
Source: Nat Commun. 2019 Jan 10;10:104. doi: 10.1038/s41467-018-08004-0 (PMC6328551; doi:10.1038/s41467-018-08004-0)
Supplement: Supplementary file 1 — Supplementary Information [file 41467_2018_8004_MOESM1_ESM.pdf]

Supplementary Information

Global Phosphoproteomic Analysis Reveals ARMC10 as an AMPK Substrate that Regulates Mitochondrial Dynamics

Chen et al.

a

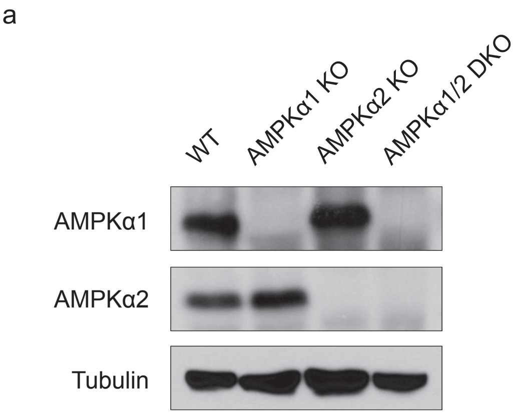

b

AMPKα1 gRNA   TCCTGTTACAGATTGTATGCAGG
PAM

WT   TCCTGTTACAGATTGTATGCAGGCCC

8 nucleotides deletion

Allele 1   TCCTGTTACAGATT - - - - - - - - - GCCC

4 nucleotides deletion

Allele 2   TCCTGTTACAGATTGT- - - - AGGCCC

AMPKα2 gRNA   ACGTTATTTAAGAAGATCCG AGG
PAM

WT   ACGTTATTTAAGA AGATCCGAGGGG

1 nucleotides insertion and 3 nucleotides deletion

Allele 1   ACGTTATTTAAGACA - - CCGAGGGG

5 nucleotides deletion

Allele 2   ACGTTATTTAAGA A - - - - - GAGGGG

c

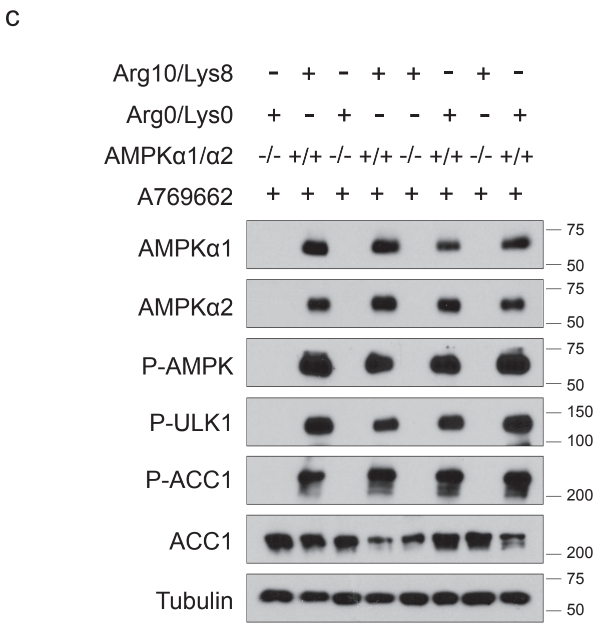

Supplementary Figure 1 AMPKα1/α2-DKO confirmation and SILAC sample quality control.

a, b) Double knockout (DKO) of AMPKα1/α2 in HEK293A cells. **(a)** Western blotting was performed to confirm that the KO target protein was not detectable. **(b)** The targeted sequence in the genome was then amplified by PCR and sub-cloned into the T vector. Twelve clones were selected for sequencing based on white-blue plaque selection. Only two mutated or deleted forms were identified from those 12 sequence results, and no wild-type (WT) sequence was recovered. PAM, protospacer adjacent motif.

c) Quality control for all four repeats in the stable isotope labeling of amino acids in cell culture (SILAC)–labeled global quantitative phosphoproteomic samples. Cells in light (Arg0/Lys0) or heavy (Arg10/Lys8) medium were treated or not treated with A769622 100 μM for 24 hours. Western blot was conducted using antibodies as indicated.

a

AMPK substrates

		-5	-4	-3	-2	-1	0	+1	+2	+3	+4
Primary Motif		L	R	R	V	X	S	X	P	N	L
Secondary Motif		M	K	K	S	X	S	X	X	D	V
Additional Motif		I	X	H	R	X	S	X	X	E	I
LARP7	S298	G	K	R	K	R	S	S	S	E	D
ARMC10_2	S45	I	R	S	S	K	S	A	G	A	L
ARMC10_1	S45	I	R	S	S	K	S	A	E	D	L
PGRMC2	S104	K	K	R	D	F	S	L	E	Q	L
AGPAT9	S68	L	K	N	S	A	S	V	G	I	I
SSFA2	S739	L	R	R	S	Q	S	L	P	T	T
KLC4	S590	M	K	R	A	A	S	L	N	Y	L
LIPE	S855	M	R	R	S	V	S	E	A	A	L
DDB2	S26	N	K	R	S	R	S	P	L	E	L
DDB2	S24	P	R	N	K	R	S	R	S	P	L
EML3	S176	S	R	K	A	I	S	S	A	N	L
LIMCH1	S217	S	R	Q	T	P	S	P	D	V	V
RAB11FIP1	S435	E	S	R	R	S	S	L	L	S	L
DHCR7	S14	I	P	K	A	K	S	L	D	G	V
REEP2	S150	K	L	R	S	F	S	M	Q	D	L
AHNAK	S135	K	P	R	L	K	S	E	D	G	V
NUFIP2	S652	L	E	R	N	D	S	W	G	S	F
MAST2	S74	L	F	R	K	L	S	N	P	D	I
ESYT2	S738	L	G	R	S	S	S	S	L	L	A
CGN	S131	L	L	R	S	H	S	Q	A	S	L
GGT7	S72	L	Q	R	L	P	S	S	S	S	E
MTFR1L	S238	L	S	K	A	S	S	F	A	D	M
MFF	S172	L	V	R	N	D	S	L	P	V	L
ABLIM1	S706	M	D	R	G	V	S	M	P	N	M
GOLGA4	S71	Q	L	R	V	P	S	V	E	S	L
REEP1	S152	R	L	R	S	F	S	M	Q	D	L
LSR	S493	R	P	R	A	R	S	V	D	A	L
NUMA1	S1969	T	L	R	R	A	S	M	Q	P	I
CTNNB1	S552	T	Q	R	R	T	S	M	G	G	T
ZFYVE16	S946	I	S	Q	V	P	S	V	E	K	L
C7orf50	S175	L	D	E	E	G	S	D	P	P	L
SMCR8	S498	L	T	V	P	L	S	P	Q	V	V

b

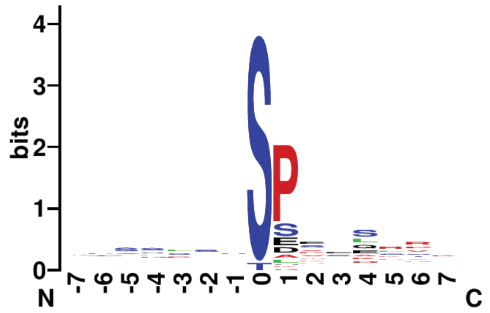

c

d

e

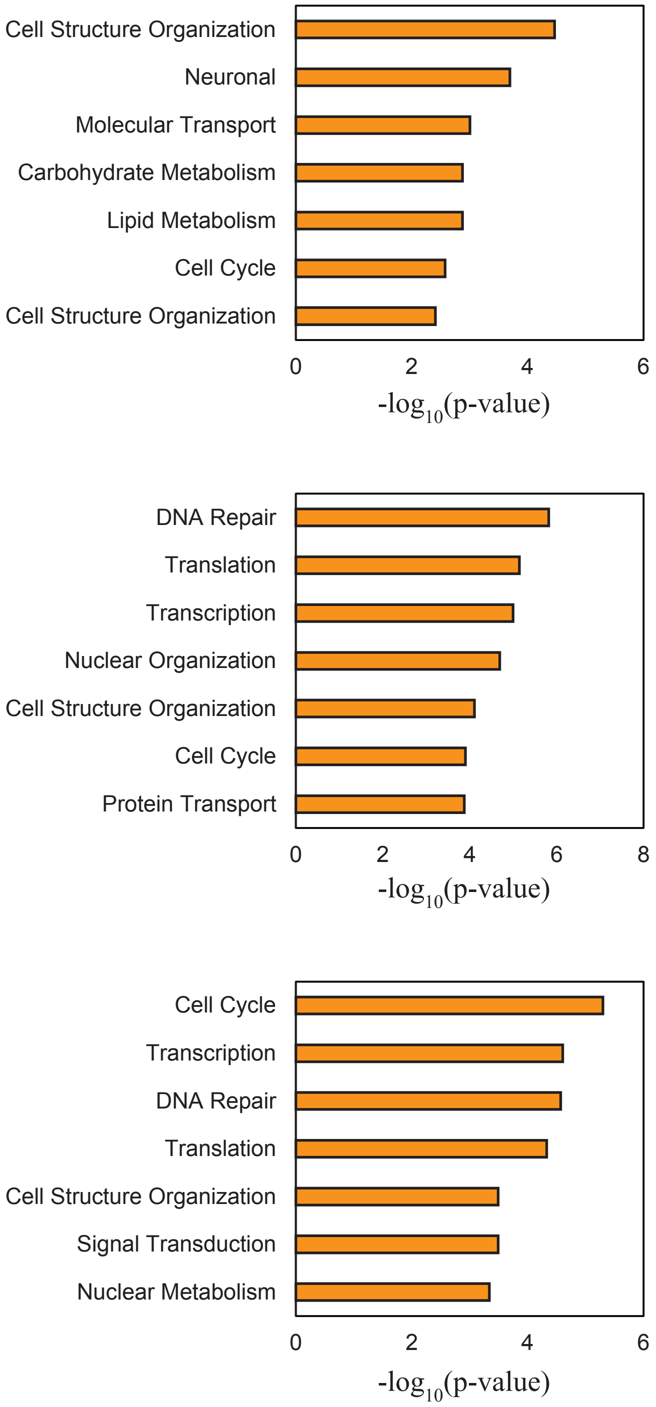

Supplementary Figure 2 Analysis of phosphoproteomics results.

a) Phosphosites and sequences in group 1, which comprised sites whose phosphorylation was significantly greater in AMPK wild-type (WT) cells than in AMPKα1/α2-DKO double knockout cells and that have peptide sequences similar to the conserved AMPK substrate motif.

b) Motif analysis of the phosphosites in group 3 (sites whose phosphorylation level was significantly lower in AMPK WT cells than in AMPKα1/α2-DKO cells) with WebLogo to create the sequence logos.

c-e) Function characterization with Ingenuity Pathway Analysis. **(c)** Group 1 (upregulated phosphosites comparing between AMPK WT cells and AMPKα1/α2-DKO cells, with the conserved AMPK substrate motif); **(d)** group 2 (upregulated phosphosites without the conserved AMPK substrate motif); and **(e)** group 3 (downregulated phosphosites).

a

Primary Motif	L	R	R	V	X	S	X	P	N	L
Secondary Motif	M	K	K	S	X	S	X	X	D	V
Additional Motif	I	X	H	R	X	S	X	X	E	I
	-5	-4	-3	-2	-1	0	+1	+2	+3	+4
ACC1 (S79)	I	R	S	S	M	S	G	L	H	L
ULK1 (S555)	F	G	R	S	F	S	T	S	R	L
RAPTOR (S792)	M	R	R	A	S	S	Y	S	S	L
MFF (S172)	L	V	R	N	D	S	L	P	V	L
Substrate Motif antibody	L	X	R	X	X	S	X	X	X	X

b

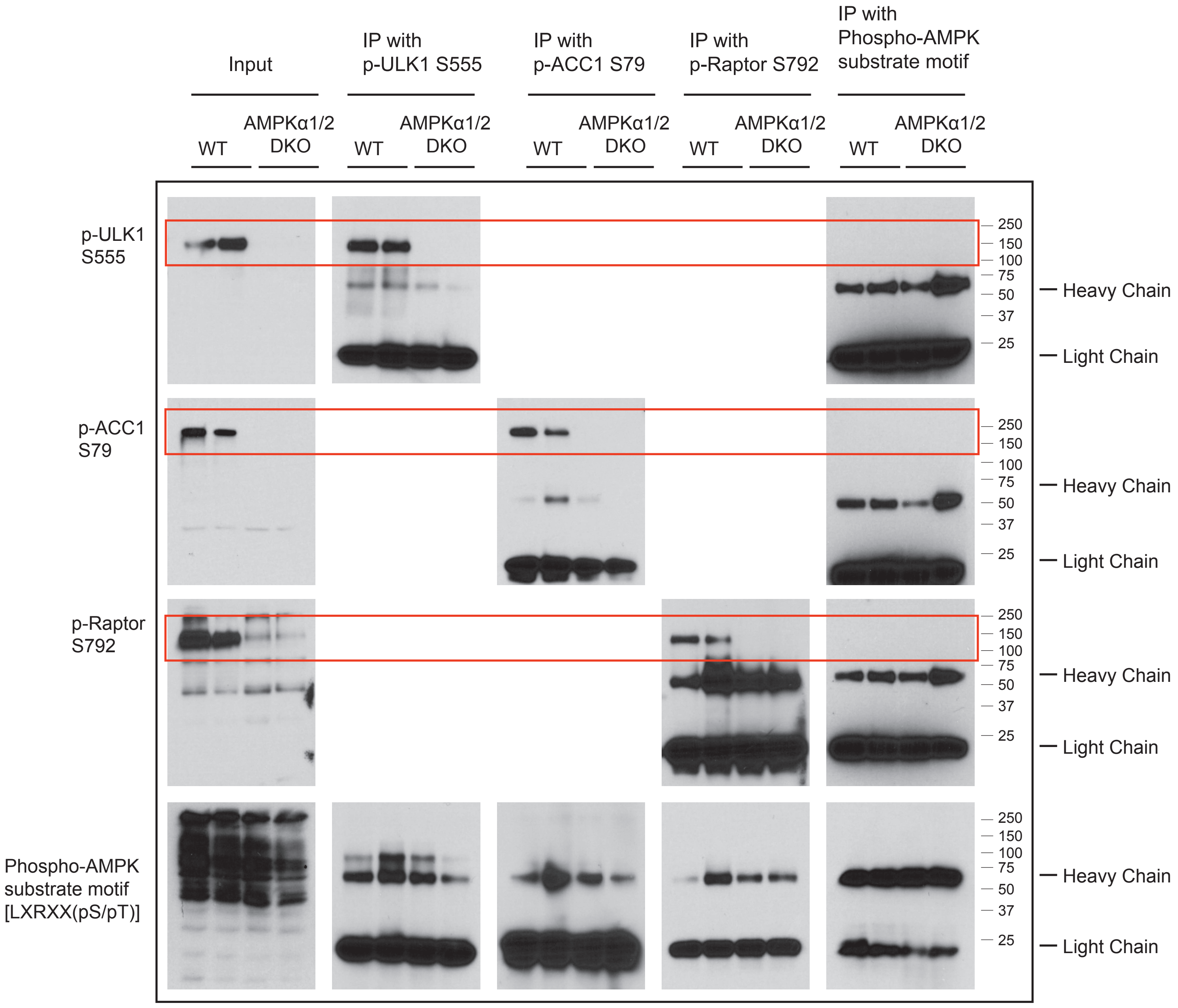

Supplementary Figure 3 Validation of the anti-phospho-AMPK substrate motif [LXRXX(pS/pT)] antibody.

a) Sequences of the three known AMPK substrates: ACC1 S79, ULK1 S555 and RAPTOR S792. In addition, the sequence of MFF S172 and the antibody-targeted sequence are shown.

b) Validation of the anti-phospho-AMPK substrate motif antibody. Samples of AMPK wild-type (WT) cells or AMPKα1/α2-DKO cells were immunoprecipitated (IP) with the indicated phospho-specific antibodies or the anti-phospho-AMPK substrate motif antibody and then blotted with the indicated phospho-specific antibodies. The red boxes are the phospho-substrate band as labeled on the left. Heavy or light immunoglobulin chains of the antibodies were labeled.

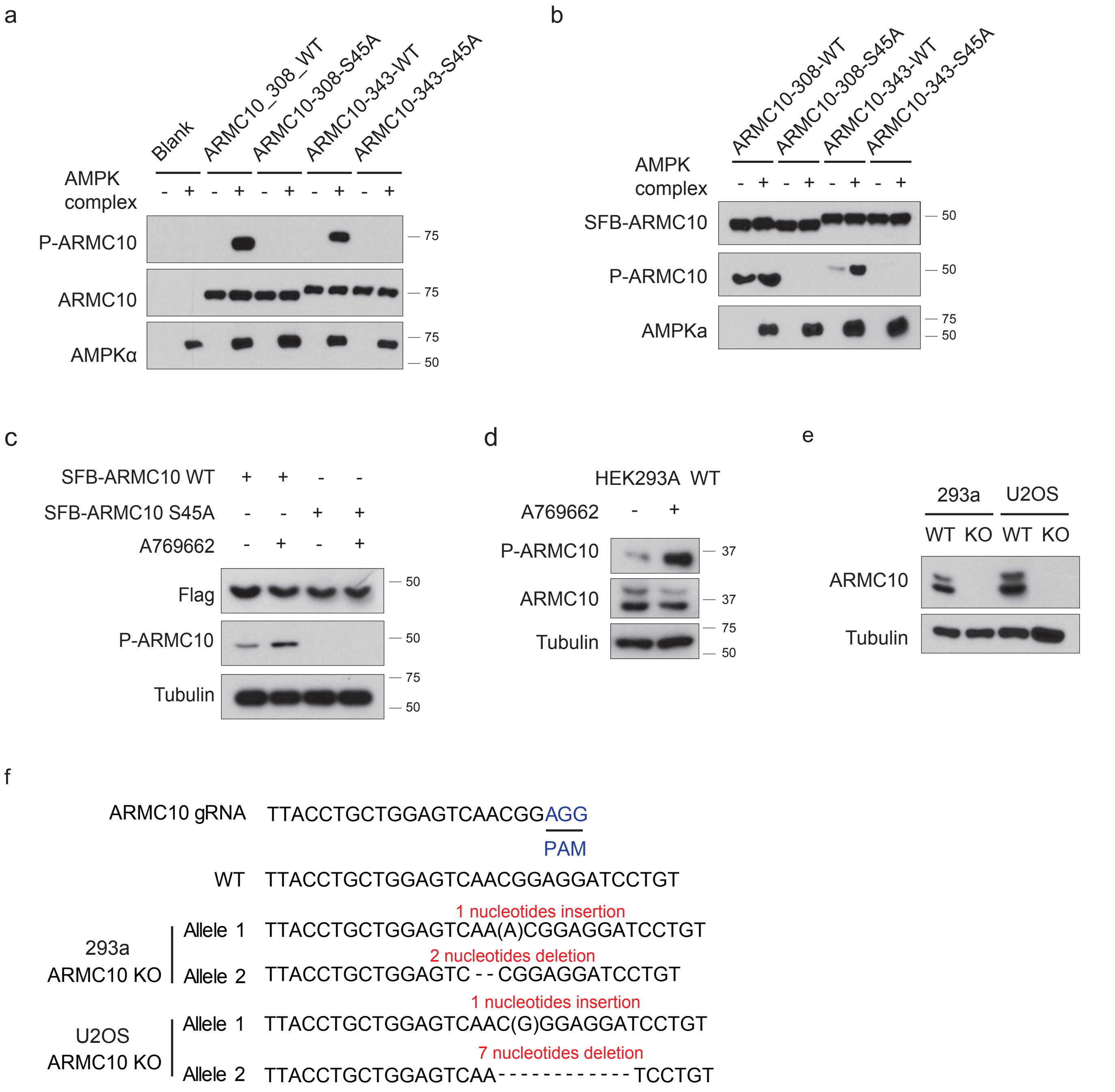
a
Blank
ARMC10_308_WT
ARMC10-308-S45A
ARMC10-343-WT
ARMC10-343-S45A
AMPK complex
- + - + - + - + - +
P-ARMC10
ARMC10
AMPKα
75
75
75
50
b
ARMC10-308-WT
ARMC10-308-S45A
ARMC10-343-WT
ARMC10-343-S45A
AMPK complex
- + - + - + - +
SFB-ARMC10
P-ARMC10
AMPKa
50
50
75
50
c
SFB-ARMC10 WT + + - -
SFB-ARMC10 S45A - - + +
A769662 - + - +
Flag
P-ARMC10
Tubulin
50
50
75
50
d
HEK293A WT
A769662 - +
P-ARMC10
ARMC10
Tubulin
37
37
75
50
e
293a
U2OS
WT KO WT KO
ARMC10
Tubulin
f
ARMC10 gRNA TTACCTGCTGGAGTCAACGGAGG
PAM
WT TTACCTGCTGGAGTCAACGGAGGATCCTGT
1 nucleotides insertion
293a ARMC10 KO
Allele 1 TTACCTGCTGGAGTCAA(A)CGGAGGATCCTGT
2 nucleotides deletion
Allele 2 TTACCTGCTGGAGTC - - CGGAGGATCCTGT
1 nucleotides insertion
U2OS ARMC10 KO
Allele 1 TTACCTGCTGGAGTCAAC(G)GGAGGATCCTGT
7 nucleotides deletion
Allele 2 TTACCTGCTGGAGTCAA- - - - - - - - - - - - TCCTGT

Supplementary Figure 4 Validation of ARMC10 phosphorylated by AMPK.

a) Both isoforms of ARMC10 were phosphorylated by the AMPK complex at the S45 site. *In vitro* kinase assays were performed without radioactivity using an anti-phospho-S45 ARMC10 antibody.

b) *In vitro* kinase assays were conducted with purified ARMC10 proteins isolated from mammalian cells to confirm that AMPK can phosphorylate WT ARMC10 but not the phosphorylation-deficient ARMC10 S45A.

c, d) *In vivo* analysis of ARMC10 phosphorylation before and after treatment with the AMPK activator A769662 100 μM for 24 hours. Phosphorylated ARMC10 was detected **(c)** in HEK293A cells in which SFB-tagged ARMC10 wild-type (WT) or S45A mutant form was overexpressed or **(d)** in WT HEK293A cells (i.e., endogenous expression) using the anti-phospho-S45 ARMC10 antibody.

e, f) KO of ARMC10 in HEK293A and U2OS cells. We selected the gRNA targeting the common region between two ARMC10 isoforms to completely delete ARMC10. Western blotting **(e)** and targeted sequencing **(f)** were used to confirm these KO cells.

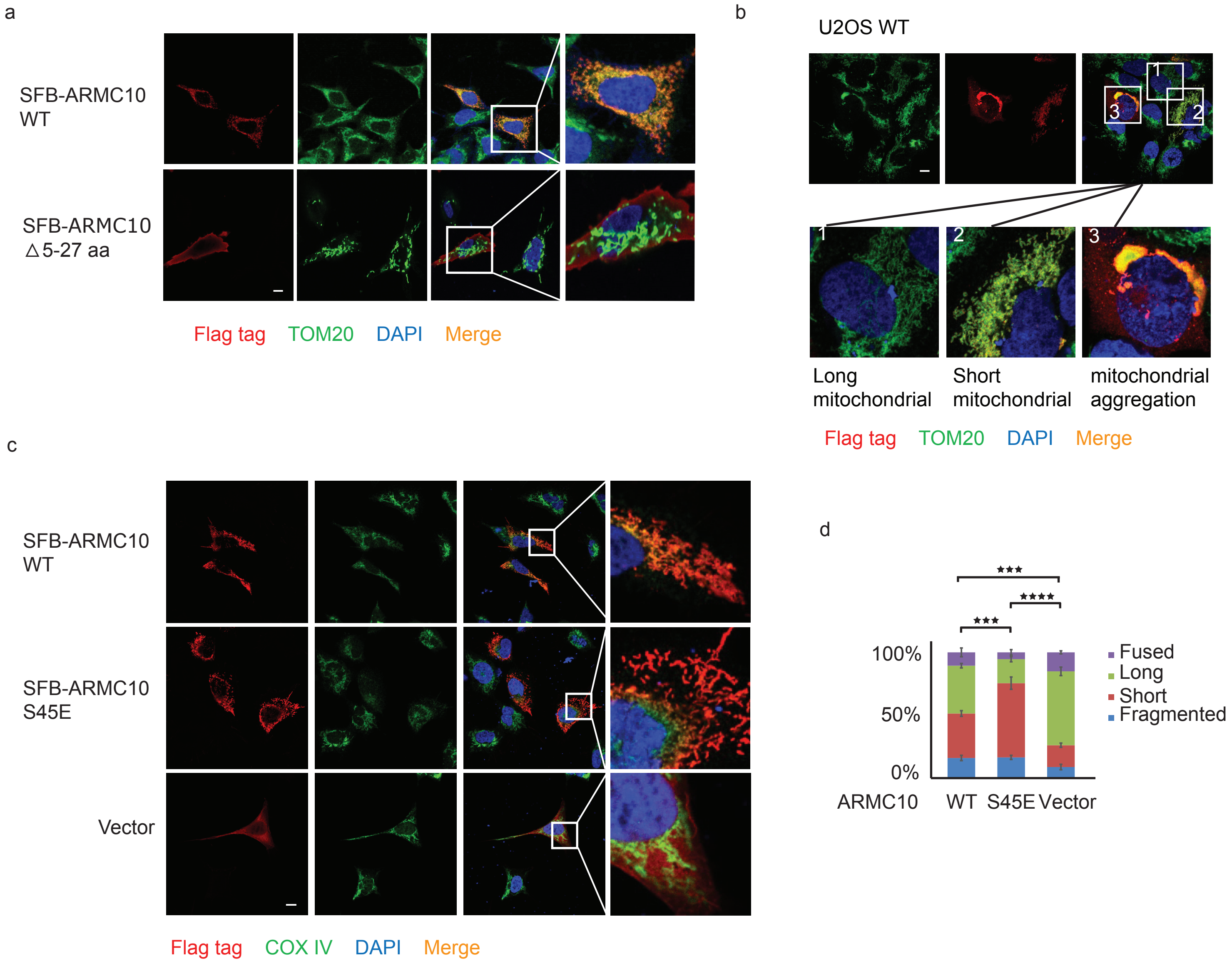
a
SFB-ARMC10 WT
SFB-ARMC10 △5-27 aa
Flag tag TOM20 DAPI Merge
b
U2OS WT
1
2
3
Long mitochondrial
Short mitochondrial
mitochondrial aggregation
Flag tag TOM20 DAPI Merge
c
SFB-ARMC10 WT
SFB-ARMC10 S45E
Vector
Flag tag COX IV DAPI Merge
d
***
****
***
100%
50%
0%
ARMC10
WT
S45E
Vector
Fused
Long
Short
Fragmented

Supplementary Figure 5 Functional study of ARMC10.

a) Analysis of the mitochondrial morphology in cells with wild-type (WT) ARMC10 or truncated ARMC10 with deletion of its transmembrane domain. The green signal indicates the mitochondrial marker TOM20, the red signal indicates anti-Flag staining for WT ARMC10 or the truncated form of ARMC10 and the blue signal indicates DAPI/nuclei. Scale bar: 10 μm.

b) Overexpression of ARMC10 may induce mitochondrial aggregation. The green signal indicates the mitochondrial marker TOM20, the red signal indicates anti-Flag staining for WT ARMC10, and the blue signal indicates DAPI/nuclei. Scale bar: 10 μm.

c, d) Immunostaining with another mitochondrial marker, Complex IV (or cytochrome c oxidase). In **(c)**, immunostaining images. The green signal indicates the mitochondrial marker Complex IV, the red signal indicates anti-Flag staining for WT ARMC10, ARMC10 S45E mutant, or vector control, and the blue signal indicates DAPI/nuclei. Scale bar: 10 μm. A region of the cell in these images was enlarged to show mitochondrial morphology. **(d)** Quantification of mitochondrial morphology in cells shown in **(c)**. Morphology was quantified as for Fig. 4b.

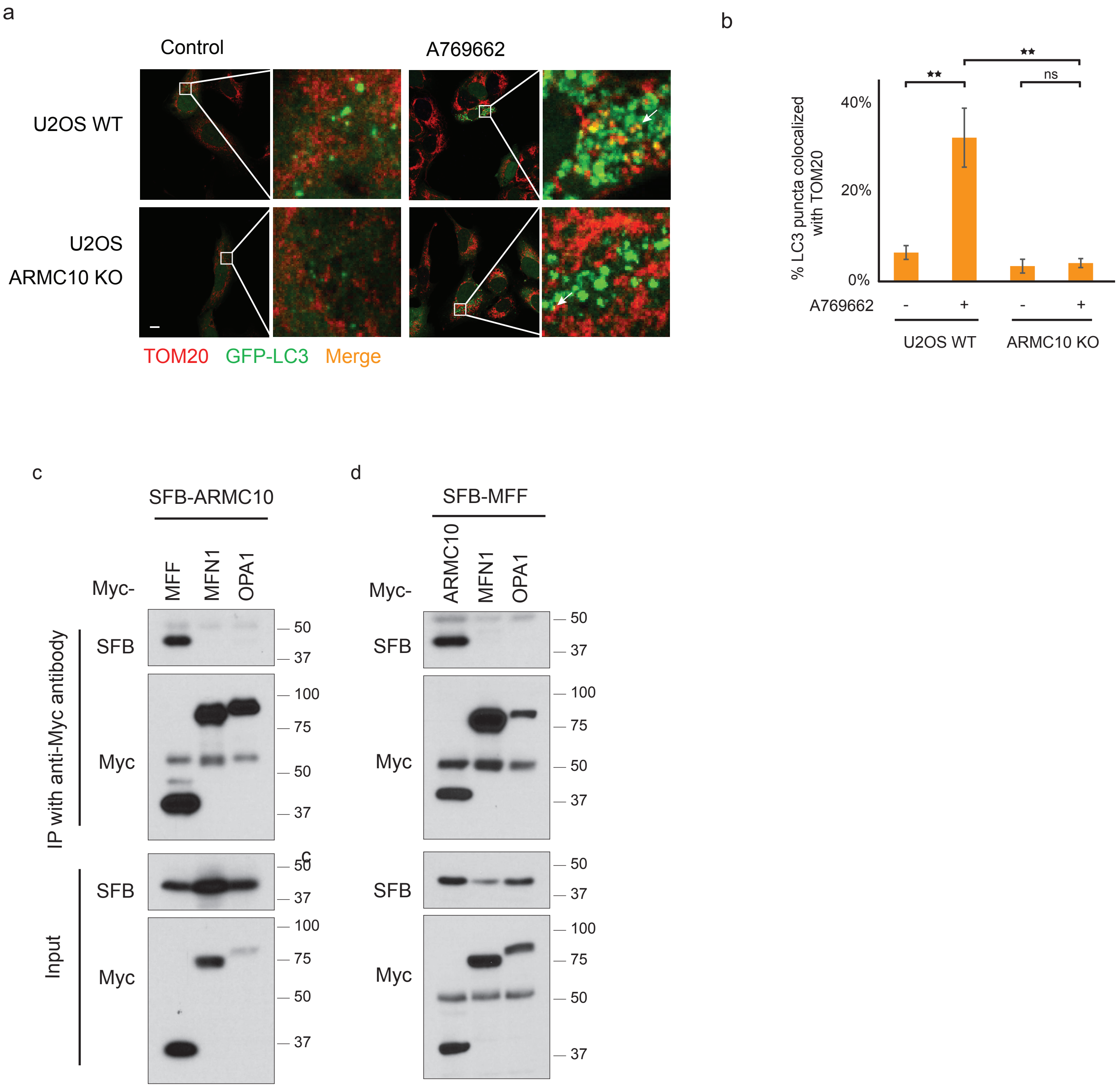

a
Control
A769662
U2OS WT
U2OS
ARMC10 KO
TOM20
GFP-LC3
Merge
b
% LC3 puncta colocalized with TOM20
40%
20%
0%
**
**
ns
A769662
-
+
-
+
U2OS WT
ARMC10 KO
c
SFB-ARMC10
Myc-
MFF
MFN1
OPA1
IP with anti-Myc antibody
Input
SFB
Myc
50
37
100
75
d
SFB-MFF
ARMC10
MFN1
OPA1

Supplementary Figure 6 Functional study of ARMC10.

a, b) Immunostains of WT and ARMC10-KO U2OS cells with green fluorescent protein (GFP)-LC3 expression. **(a)** The green signal indicates (GFP)-LC3, and the red signal indicates the mitochondrial marker TOM20 stained with TOM20 antibody. The cells were treated or not treated with AMPK activator A769662 300 μM for 4 hour. The white arrows are examples of colocalizated LC3 puncta and TOM20. Scale bar: 10 μm. **(b)** The quantification graph shows the percentage of colocalization puncta of GFP-LC3 and TOM20 per cell. It was quantified from more than 20 cells with puncta staining per condition (mean ± standard deviation; n=3 biologically independent extracts). **p<0.01 (Student's *t*-test); ns, not significant.

c, d) ARMC10 interacts with MFF. To test if ARMC10 binds to MFF, we conducted an immunoprecipitation (IP) experiment between these two proteins and included MFN1 and OPA1 as negative controls. Constructs encoding indicated tagged proteins were transfected into HEK293T cells. Cells were collected 24 hours later and cell lysates were subjected to pulldown assay with anti-Myc antibody and Protein A agarose beads. Western blotting was conducted using indicated antibodies. **(c)** The binding between SFB-tagged ARMC10 and Myc-tagged MFF, MFN1 or OPA1. **(d)** The binding between SFB-tagged MFF and Myc-tagged ARMC10, MFN1 or OPA1.

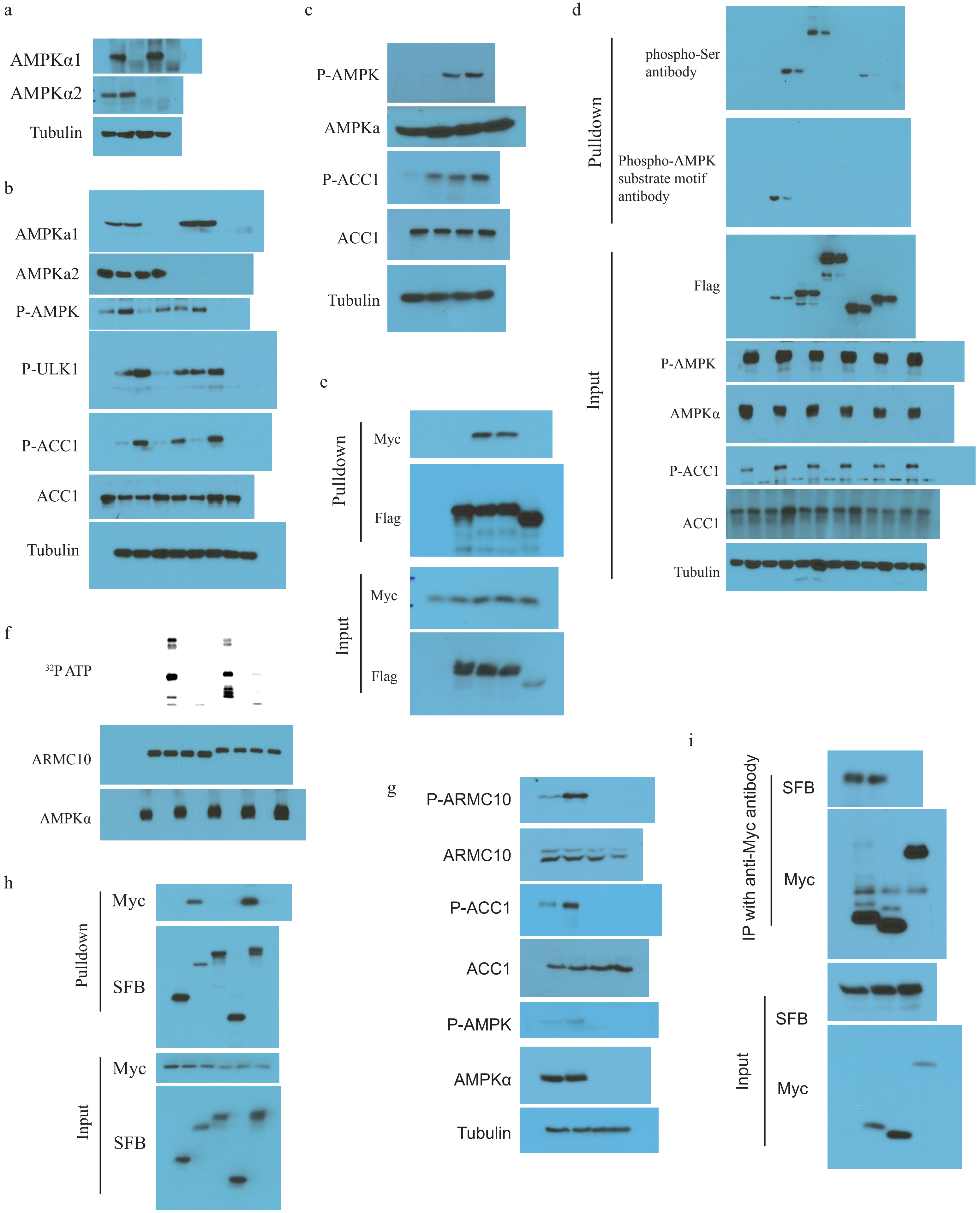
a
AMPKα1
AMPKα2
Tubulin
b
AMPKa1
AMPKa2
P-AMPK
P-ULK1
P-ACC1
ACC1
Tubulin
c
P-AMPK
AMPKa
P-ACC1
ACC1
Tubulin
d
Pulldown
phospho-Ser antibody
Phospho-AMPK substrate motif antibody
Input
Flag
P-AMPK
AMPKα
P-ACC1
ACC1
Tubulin
e
Pulldown
Myc
Flag
Input
Myc
Flag
f
32P ATP
ARMC10
AMPKα
g
P-ARMC10
ARMC10
P-ACC1
ACC1
P-AMPK
AMPKα
Tubulin
h
Pulldown
Myc
SFB
Input
Myc
SFB
i
IP with anti-Myc antibody
SFB
Myc
Input
SFB
Myc

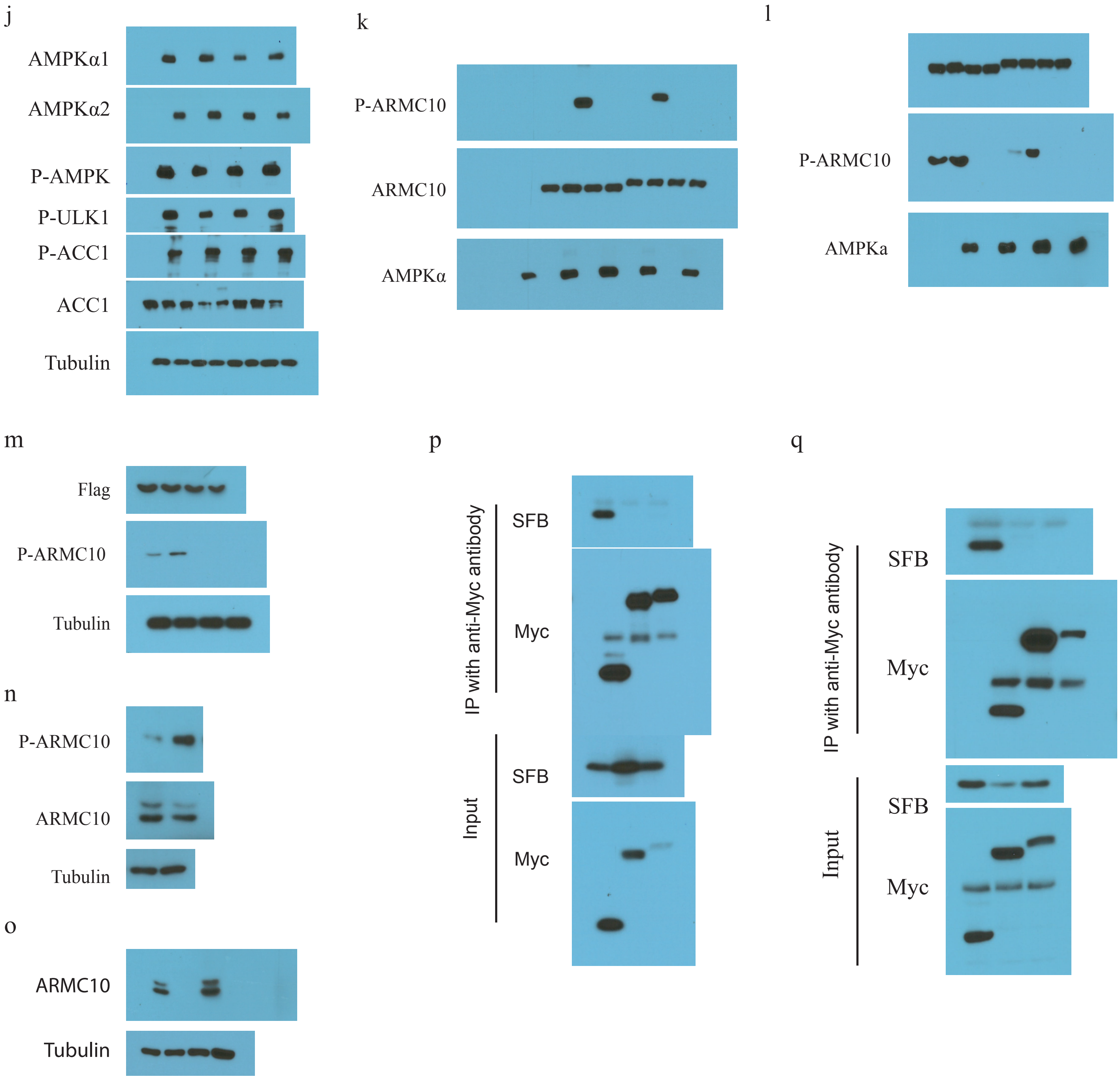
j
AMPKα1
AMPKα2
P-AMPK
P-ULK1
P-ACC1
ACC1
Tubulin
k
P-ARMC10
ARMC10
AMPKα
l
P-ARMC10
AMPKa
m
Flag
P-ARMC10
Tubulin
n
P-ARMC10
ARMC10
Tubulin
o
ARMC10
Tubulin
p
IP with anti-Myc antibody
SFB
Myc
Input
SFB
Myc
q
IP with anti-Myc antibody
SFB
Myc
Input
SFB
Myc

Supplementary Figure 7 Uncropped Western blot scan.

a) uncropped western blot scan from Fig. 1a and Supplementary Figure 1a;
b) uncropped western blot scan from Fig. 1b;
c) uncropped western blot scan from Fig. 1c;
d) uncropped western blot scan from Fig. 3b;
e) uncropped western blot scan from Fig. 3d;
f) uncropped western blot scan from Fig. 3e;
g) uncropped western blot scan from Fig. 3f;
h) uncropped western blot scan from Fig. 6b;
i) uncropped western blot scan from Fig. 6c;
j) uncropped western blot scan from Supplementary Figure 1b;
k) uncropped western blot scan from Supplementary Figure 4a;
l) uncropped western blot scan from Supplementary Figure 4b;
m) uncropped western blot scan from Supplementary Figure 4c;
n) uncropped western blot scan from Supplementary Figure 4d;
o) uncropped western blot scan from Supplementary Figure 4e;
p) uncropped western blot scan from Supplementary Figure 6c;
q) uncropped western blot scan from Supplementary Figure 6d.

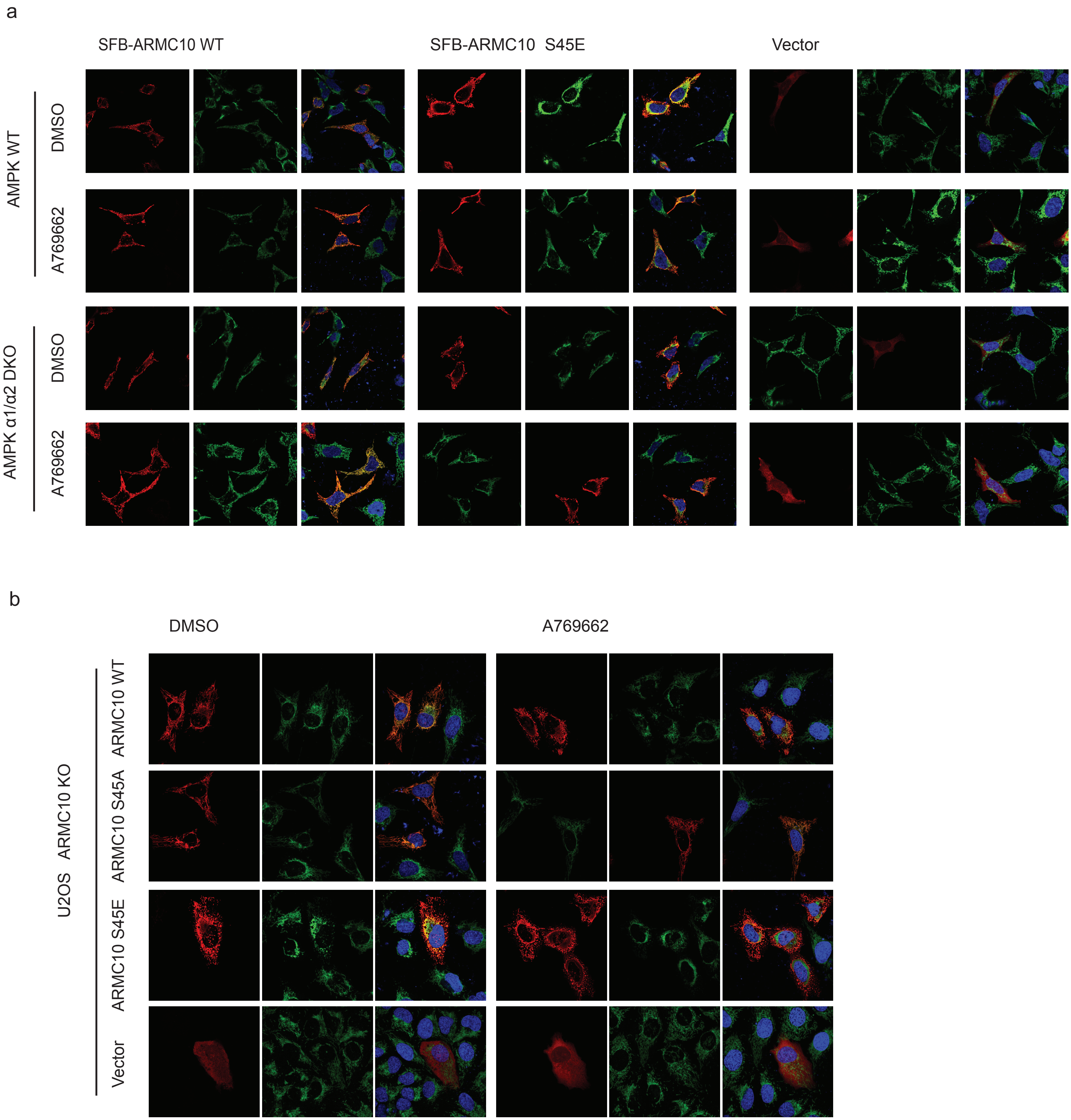
a
SFB-ARMC10 WT
SFB-ARMC10 S45E
Vector
AMPK WT
DMSO
A769662
AMPK α1/α2 DKO
DMSO
A769662
b
DMSO
A769662
U2OS ARMC10 KO
ARMC10 WT
ARMC10 S45A
ARMC10 S45E
Vector

Supplementary Figure 8 Unmerged immunostaining images.

a) unmerged Immunostaining images from Fig 4c;
b) unmerged Immunostaining images from Fig 5a.
